# The Effect of Fullerene Soot Nanoparticles on the Microstructure and Properties of Copper-Based Composites

**DOI:** 10.3390/nano10101929

**Published:** 2020-09-27

**Authors:** Elizaveta V. Bobrynina, Tatiana V. Larionova, Tatiana S. Koltsova, Aleksey I. Shamshurin, Oksana V. Nikiforova, Oleg V. Tolochko, Ji Puguang, Yin Fuxing

**Affiliations:** 1 Peter the Great Saint-Petersburg Polytechnic University, Politekhnicheskaya 29, 195251 St. Petersburg, Russia; larionova@hotmail.com (T.V.L.); annelet@yandex.ru (T.S.K.); sham_a@mail.ru (A.I.S.); tolochko_ov@spbstu.ru (O.V.T.); 2 The National Technological Initiative Competence Center of SPbPU, Politekhnicheskaya 29, 195251, St. Petersburg, Russia; oksana-nikiphorova@yandex.ru; 3 Research Institute for Energy Equipment Materials, Hebei University of Technology, Tianjin 300130, China; jipuguang@hebut.edu.cn (J.P.); wang.gongkai@hebut.edu.cn (Y.F.); 4 School of Material Science & Engineering, Hebei University of Technology, Tianjin 300130, China; 5 Tianjin Key Laboratory of Materials Laminating Fabrication and Interface Control Technology, Hebei University of Technology, Tianjin 300130, China

**Keywords:** copper matrix composite, fullerene soot, ball milling, microstructure, strengthening

## Abstract

Copper-based composite materials strengthened with nanosized fullerene soot particles were produced by mechanical milling and hot pressing technology with a content of carbon up to 5 wt. %. The microstructure of the composite powders and the compacts prepared using them were examined by light microscopy, SEM, EDS, XRD, and XPS; hardness, heat conductivity, and tribological characteristics were measured. The interesting feature of the observed microstructure was a “marble” pattern formed by a white boundary net. The study shows homogeneous distribution of carbon inside the copper grains and its lower concentration in the grain boundaries. The effect was caused by a reaction of carbon with oxygen adsorbed by the copper particles surface. The maximal hardness of the material is 160 HB for the sample with 0.5 wt. % of fullerene soot; this material has the minimal friction coefficient (0.12) and wear in a dry friction condition. Heat conductivity of the material (Cu-0.5 wt. % C) is 288 W/m*K.

## 1. Introduction

Copper-based composites reinforced with carbon nanoparticles attract interest due to a combination of mechanical and electrical properties [1,2,3,4,5,6,7,8,9,10,11,12,13,14,15,16,17,18,19,20,21,22,23]; besides, these composites mostly have low friction coefficient and wear [14,15,16,17,18,19], which is necessary for sliding contact application.

The strengthening of Cu-C composites in general is determined by the amount and type of carbon added, as well as the homogeneity of its dispersion and bonding to the matrix. The latter primarily depends on the method of introducing carbon particles into the metal matrix. The dominant mechanism of strengthening in the majority of Cu-C composites is a load transfer, and this mechanism is essential only when the reinforcing phase has a very high strength and stiffness. On the other side, strengthening may be achieved via hindering the dislocation glide in the ductile matrix by different barriers, such as grain boundaries, disperse particles, stress caused by differences in thermal expansion coefficients of matrix and second phase, and other barriers. It is widely known that the strength of a material depends on its grain size, and the effect is described by the Hall–Petch equation σ = σ_0_ + Kd^-1/2^. The Hall–Petch effect is considered a main strengthening effect in a number of Cu-C composites [12,14]. It is especially important in the composites where carbon nanostructures have been grown on the Cu particles’ surface [6,14]. From this point of view, any carbon nanosized particles can be utilized as a reinforcement phase in a copper matrix, if they are well distributed and have a good interfacial bonding with the matrix. As a reinforcement phase, carbon nanotubes (CNTs) have gained the greatest attention due to their high mechanical and physical properties, caused by their unique structure [1,2,3,4,5,6,7,8,9,10,11,12,13,14,15,16,17,18,19]. Thus far, a number of research papers devoted to graphene oxide in copper-based composites have appeared [20,21,22,23]. One of the reasons of the ascending interest is the lower cost and the availability of the latter. Fullerenes were recently successfully used in composites on the base of aluminum [24,25,26] and magnesium [27]. However, to date, Cu-based composites with fullerene additions are barely discussed in the literature. In the recent work in [28], the influence of fullerene additions on structure and phase composition of Cu-C composite powders was discussed, but properties of compacted materials were not studied. Another type of carbon material, which may be considered a reinforcement to Cu-based composites, is fullerene soot (FS). As reported in [29], combustion of fullerene soot mainly consists of a mixture of spherical particulate carbons of a size of about 30 nm with a very narrow size distribution. With the exception of these very fine particles, the soot contains fullerene structures and occasionally observed large crystals of carbon black [29]. These characteristics—very small size, round shape, and narrow size distribution—might be prospective factors in order to use the soot as a phase for dispersion strengthening. In addition, it is significantly cheaper than other nanocarbon materials, which makes it attractive for composite production. Up to now, there have been no works devoted to Cu-based composites reinforced with fullerene soot.

In this study, Cu-based composites of full density with varied FS content were prepared by mechanical milling and hot pressing. The evolution of the microstructure and especially the processes in the Cu-C interphase during preparation were analyzed. The effects of FS on hardness and thermal conductivity of the Cu-based composites were evaluated.

## 2. Materials and Methods

**Figure 1:**
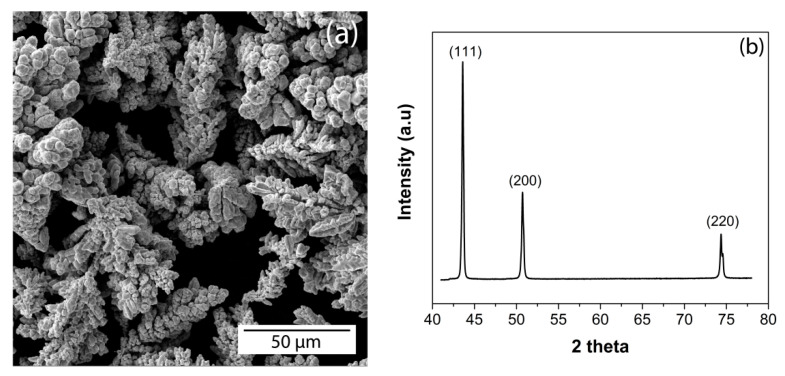
SEM micrograph (**a**) and XRD (**b**) of initial copper powder.

**Figure 2:**
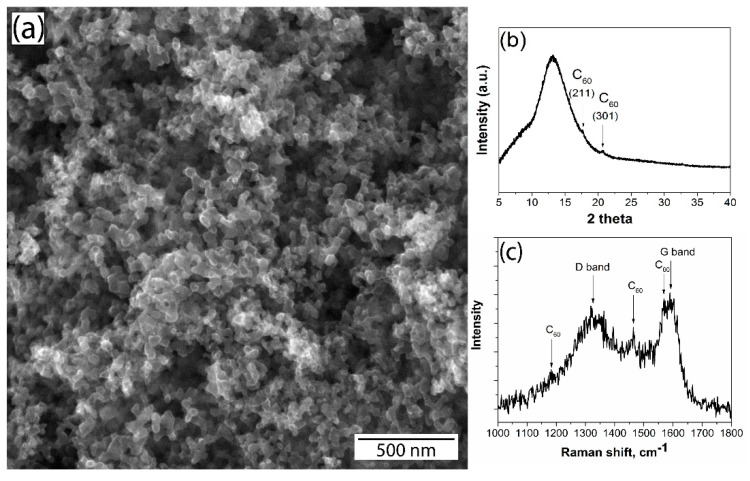
SEM micrograph (**a**), XRD (**b**), and Raman spectra (**c**) of the initial fullerene soot.

Copper matrix composites were produced by powder metallurgy route. The initial copper powder is a commercial powder of PMS1 grade (GOST 4960–2009, HimSnab, Saint-Petersburg, Russia) of 99.5% purity produced by the electrolytic method. The Cu particles were dendrite crystals with an average size of 40 μm, as shown in Figure 1. Fullerene soot (FS) was supplied by Suzhou Dade Carbon Nanotechnology Co (Suzhou, China). As claimed by the supplier, the content of fullerenes in the soot was about 11–13%. As seen on the SEM image, as shown in Figure 2a, FS particles have a spherical shape and a size of 20–40 nm. As revealed by XRD, as shown in Figure 2b, and Raman spectroscopy, as shown in Figure 2c, the FS consists of mostly amorphous carbon. There are very small reflexes corresponding to fullerenes observed as well. 

The copper and FS were mixed giving a FS content ranging from 0.25 to 5 wt. %. Ten grams of powder mix was put in a stainless steel jar of 80 mL volume filled with milling balls of 5 mm diameter; the ball to powder ratio was 8:1. Milling was performed in an argon atmosphere in a planetary ball mill (Pulverisette 7, Fritsch, Idar-Oberstein, Germany) in two stages. The first low energy stage was conducted at 200 rpm for 60 min and the second—high energy—at 600 rpm for 60 min as well. 

The milled powders were cold pressed at 400 MPa, then heated up to 750 °C and hot pressed at 200 MPa. For reference, samples of pure copper and Cu-5 wt. % graphite composite were prepared via the same technological route. The compacted samples were cylinders of 40 mm diameter and about 4 mm in height.

For microstructure observation, the specimens were prepared through conventional mechanical polishing. Scanning electron microscopy (SEM) and energy dispersion spectroscopy (EDS) were performed on a MIRA 3 microscope (TESCAN, Brno, Czech Republic) at a voltage of 20 kV. For light microscopy observations, the specimens were etched in a mixed FeCl_3_/HCl solution. The observations were performed with a Carl Zeiss Observer D1m microscope (Carl Zeiss, Oberkochen, Germany). The X-ray diffraction (XRD) analysis was performed using a Bruker D8 Advance diffractometer (Bruker, Billerica, MA, USA) in Cu monochromatic *K_α_* radiation. The chemical state of the elements was studied by X-ray photoelectron spectroscopy (XPS) with a Thermo Scientific K alpha spectrometer (Waltham, MA, USA); the analyzing spot was 400 μm. Differential thermal analysis (DTA) and thermal-gravimetric analysis (TGA) were conducted on a Setsys Evolution-1750 (SETARAM, Caluire-et-Cuire, France) using 30 mg of powder at a heating rate of 10 °C/min up to 900 °C in air.

Brinell hardness was tested with ZWICK ZHU (ZWICK, Ulm, Germany) under a load of 100 N and a dwelling time of 10 s. The thermal diffusivity (*α)* of the specimens was measured by the flash method using a DXF-200 (TA-Instruments, Delaware, USA). The thermal conductivity was calculated from the measured values of the thermal diffusivity by the equation *λ = αρc_p_*, where *c_p_* [J(g*K)] is the specific heat capacity of the specimen. The friction testing was performed at a load of 10–50 N by pin on plate scheme with a pin of 20 mm diameter. Wear was measured as a diameter of wear spots using rotation of a steel ball on a plate; the ball diameter was 12.7 mm. The testing was performed at a rotation speed of 200 rpm and force of 145 N during 60 s.

## 3. Results and Discussion

### 3.1. Cu-FS Composite Powders

**Figure 3:**
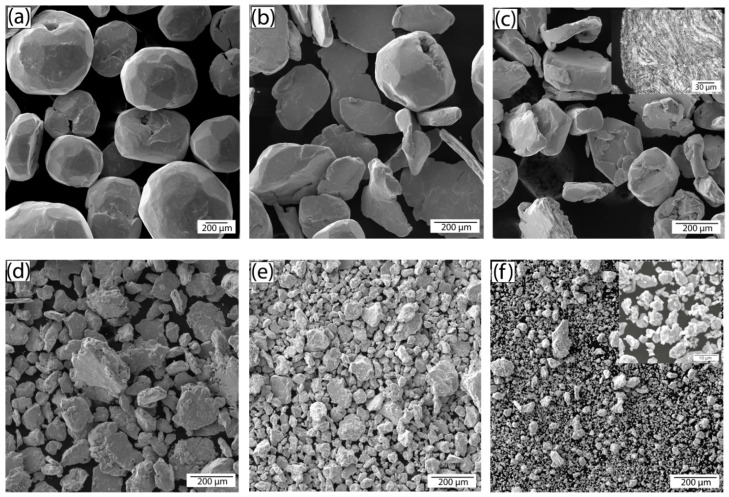
SEM images of the powders after milling: (**a**) Cu, (**b**) Cu-0.25 wt. % FS, (**c**) Cu-0.5 wt. % FS, (**d**) Cu-0.1 wt. % FS, (**e**) Cu-2 wt. % FS, (**f**) Cu-5 wt. % FS. Inset of (**c**,**f**) is a cross section of the particle (optical image) and particles with 5% of fullerene soot (FS) at high magnification.

**Figure 4:**
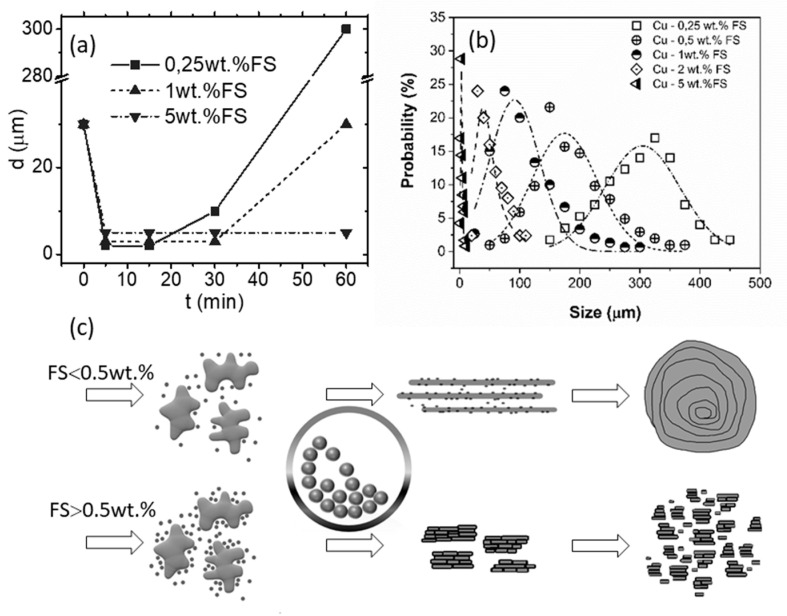
The dependence of the average flake thickness on the milling time (**a**), the resultant particle size distribution in dependence on the content of FS (**b**), and the scheme of the process (**c**).

Figure 3 shows SEM images of the powders after 60 min of milling. The change of the flakes’ thickness in dependence on the milling time and the resultant particle size distribution in dependence on the carbon content are shown in Figure 4a,b, correspondingly. As seen, an increase of FS content leads to a decrease in the size of the composite particles. 

The powders with 0.25–0.5 wt. % of carbon exhibit the behavior typical for ductile materials—milling causes the particles to flatten, then the flakes overlap and cold weld. This leads to a formation of layered particles, as shown in the inset of Figure 3c, in which carbon nanoparticles are uniformly dispersed inside the copper matrix. If the content of FS exceeds 0.5 wt. %, it completely covers the Cu surface preventing welding, so the dendrites are flattened and broken up without consolidation; carbon remains on the copper surface or hammered into it. The scheme of the process is presented in Figure 4c.

**Figure 5:**
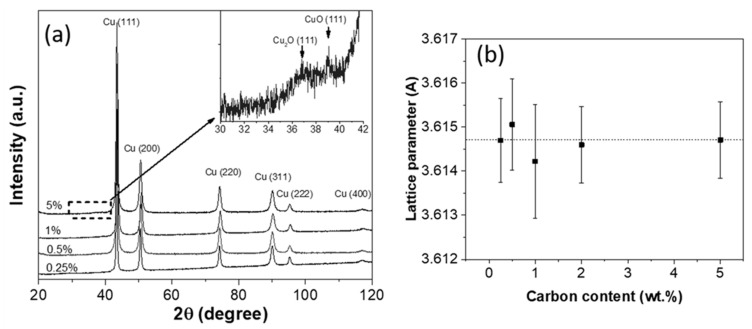
The XRD results (**a**) and copper lattice parameters (**b**) of the composite powders depending on FS content (dashed line corresponds to a reference value for pure Cu).

The prepared composite powders were analyzed by XRD, and the results are shown in Figure 5. According to the Cu-C equilibrium phase diagram, carbon does not dissolve in copper lattice; however, in [14] when the high energy ball milling of copper with fullerenes had been studied, the authors reported a formation of supersaturated solid solution Cu(C, O), which caused a growth of the Cu lattice parameter. In contrast to that, we did not detect the growth of the Cu lattice parameter for any of the samples, as shown in Figure 5b, possibly because of insufficient milling energy. We observed only the peaks broadening caused by microstrains and the crystalline size decrease; the effect is stronger for the samples with a higher carbon content. Besides, there were found weak peaks belonging to copper oxides, as shown in the inset of Figure 5, which are more pronounced in the Cu-5% FS pattern. Obviously, during milling, the specific surface of the particles is increased and becomes very active, which results in the surface oxidation either during milling (even in a protective atmosphere) or when the powders are exposed to air. The more developed surface of the powders with a higher carbon content (Cu-5% FS) leads to a greater oxidation.

### 3.2. Microstructure of Cu-FS Compact Composites

**Figure 6:**
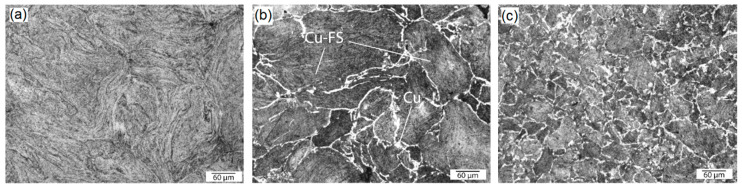
Optical images of the samples’ microstructures after hot pressing: Cu-0.25 wt. % FS (**a**), Cu-1 wt. % FS (**b**), Cu-2 wt. % FS (**c**).

**Figure 7:**
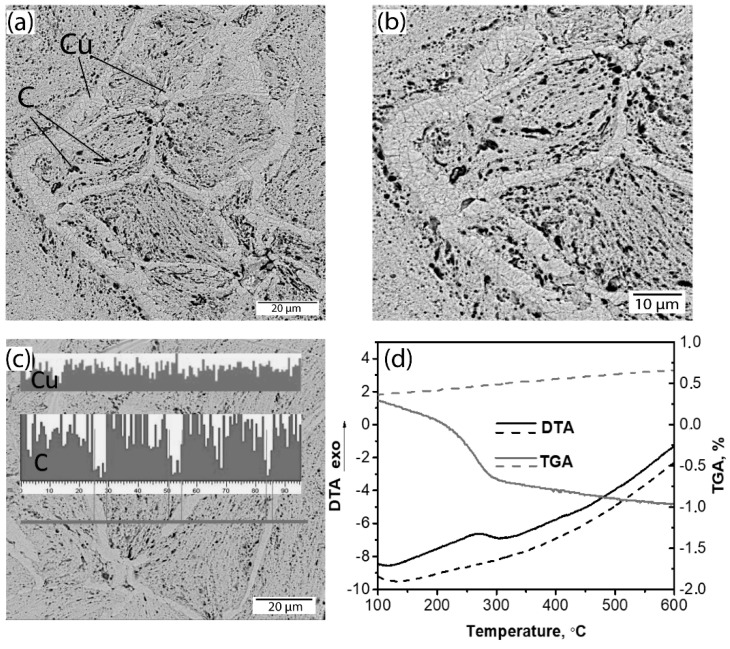
SEM images of Cu-2% FS composites (**a**–**c**). (**a**) shows visible microstructure and (**b**) is the same structure at the higher magnification, which shows the microstructure of the white boundaries. Inset of (**c**) shows the Cu and C distributions along the grey line by EDS. (**d**) shows differential thermal analysis (DTA) and TGA curves of Cu-5 % FS (solid line) and pure copper (dashed line) powders.

The microstructures of the compacts are shown in Figure 6 and Figure 7. All the samples were dense and there was no noticeable porosity observed by optical microscopy. The measurements of hydrostatical density of the composites and the estimation of relative density show that the density of the compacted composites depends on the carbon content. The dependence has a maximum (98.2%) at 0.5 wt. % of FS, and apparently, the small addition of FS (0.25 and 0.5 wt. %) led to a better densification compared even to pure copper; the same phenomenon was observed in [5,16]. FS in a concentration over 0.5 wt. % hampers metal contact between the particles and worsens sintering, leading to the relative density decreasing at 2 and 5% of FS content. However, it should be noted that the calculated relative densities are likely lower than real values, as the theoretical density was calculated without taking into account the oxide phases, as shown in Figure 5a.

An interesting feature of the observed microstructure is a “marble” pattern formed by a white boundary net, which was not observed in the pure Cu and in the sample with 0.25 wt. % FS, as shown in Figure 6a. It should be noted that these observed boundaries, in fact, are the surfaces of the as-milled composite particles, and the “seeming” grains actually consist of fine “real” copper grains (indistinguishable in the microstructures) and FS particles. EDS analysis has completely excluded the contamination of the particle surfaces due to the preparation process—the content of Fe and other possible contaminations were negligible.

The SEM image of the Cu-2 wt. % FS is presented in Figure 7a. As the image was made in the mode of back scattering electrons, it reflects the phase contrast—the grains consist of thin light layers of copper and dark spots of carbon distributed between them. The grain boundaries are light, dense, have no carbon inclusions, and consist of small equiaxial copper grains under 2 µm. In Figure 7c, the EDS shows Cu and C distributions along a scanning line; it proves that the boundaries have lower carbon concentration compared to that inside of grains, whereas copper fluctuations are independent of the location.

We assume that the decreased carbon concentration may be explained by an interaction of FS with surface copper oxide and the formation of volatile carbon containing product, which is removed through open pores during hot pressing. Reduction of the copper oxide by carbon during milling has been described in [30] and was observed in [28] as well. The reduction induces copper recrystallization, which explains the microstructure of the boundaries. 

Thermal analysis of the milled powders was conducted to verify the assumption about carbon and surface copper oxide reactions during hot pressing, as shown in Figure 7d. DTA and TGA curves for composite Cu-5 wt. % FS powder are compared to those of pure copper. DTA of the composite sample shows an exothermic effect (150–300 °C), which is accompanied by weight loss on TGA. In contrast, heating of the copper powder is accompanied by continuous weight growth due to gradual oxidation; no thermal effects were found. The milled composite powder in its initial state and after exothermic transformation were analyzed by XPS. 

**Figure 8:**
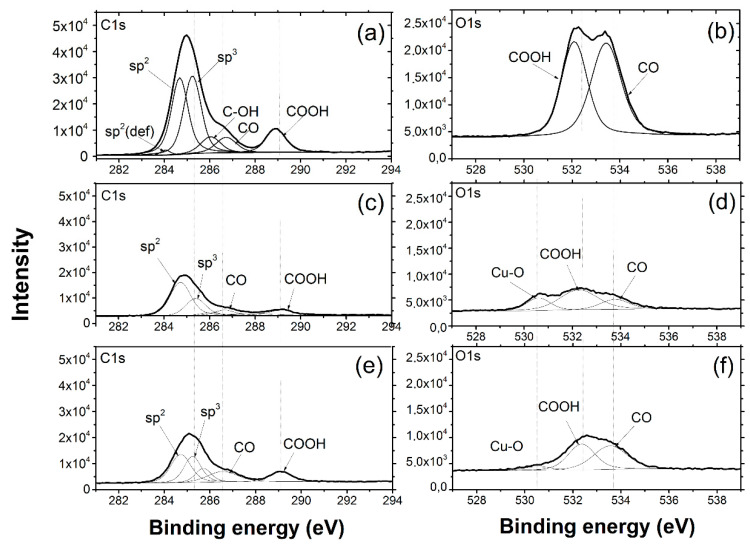
C1s (**a**–**e**) and O1s (**b**–**f**) high resolution spectra of FS (**a**,**b**), and composite Cu-5 wt. % FS powder after milling (**c**,**d**) and annealing at 400 °C (**e**,**f**).

Figure 8 shows C1s and O1s high resolution core level XPS spectra for FS itself, for just milled composite powder, and for the powder annealed at 400 °C. C1s XPS spectra of the FS clearly demonstrates the main C-C peak comprising sp^2^ (284.8 eV) and sp^3^ (285.3 eV) and additional peaks belonging to several functional groups: carboxyl (-COOH) at 289 eV, ether (-OC) at 287 eV, and hydroxyl (-OH) at 286 eV [31,32]. These groups are confirmed by the O1s spectrum observation, as it has corresponding components at 532 eV (-COOH) and at about 533.5–534 eV (-OC, OH). After milling with copper, the groups’ peaks slightly decreased the O1s spectrum showed, and besides -COOH and -OC components, a new prominent peak at 530.5 eV corresponding to O bounded to Cu. 

As seen in Figure 8e,f, annealing caused a considerable degradation of the Cu-O component. These observations have confirmed the statement proposed earlier about surface copper oxide reduction by fullerene soot during the hot pressing procedure.

It may be proposed that the reduction is carried out via interaction of Cu_2_O with both carbon and functional groups according to the reactions (1) and (2)–(3), correspondingly:

2Cu_2_O + C = CO_2_ + 4Cu (1)

Cu_2_O + 2(-COOH) = 2CO_2_ + H_2_O + 2Cu (2)

Cu_2_O + (-OC) = CO_2_ + 2Cu (3)

Apparently, it is the reduction of the surface oxide layer that results in the better compaction of the composite with 0.25–0.5 wt. % FS compared to the pure copper powder, as shown in Figure 6 (even though we have not observed the recrystallized boundaries in the Cu-0.25 wt. % FS sample). However, the formation of the gaseous product may be the reason of the increased porosity at higher FS concentrations.

**Figure 9:**
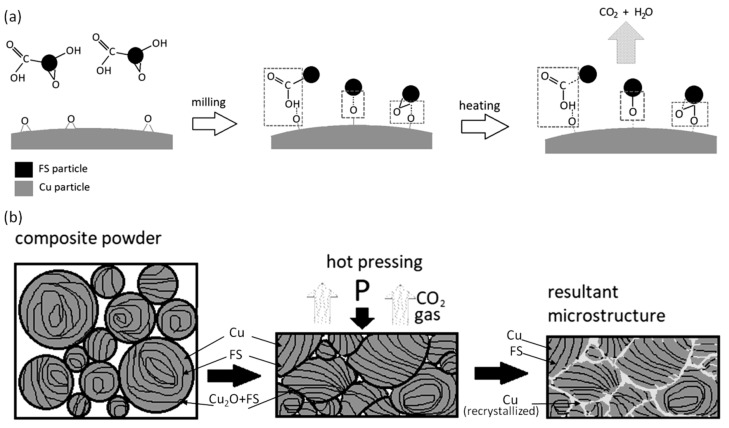
Illustration of Cu-O-C complex formation and copper reduction during milling and heating (**a**) and process of “marble” pattern formation (**b**).

Thus, during milling, FS particles are adsorbed on the copper surface via coordination of functional groups to O bound to Cu and the formation of the Cu-O-C complex, as shown in Figure 9a [32,33]. During hot pressing, the complex decomposes resulting in copper reduction and the volatilization of the carbon containing gas, as shown in Figure 9a,b. The latter leads to the weight loss observed at DTA, as shown in Figure 7d, and a depletion of the boundaries with carbon, as shown in Figure 7a. According to the proposed scheme, the functional groups promote the better adsorption of FS particles on the surface of copper flakes during milling and, as a result, promote the better distribution of them in the composite particle. The role of oxygen in the formation of the Cu-C interface was noticed in [10,34], and the authors concluded that the oxygen atoms chemically bonded to the carbon nanotube and metal interface providing strong bonding between them. 

### 3.3. Properties of the Composites 

**Figure 10:**
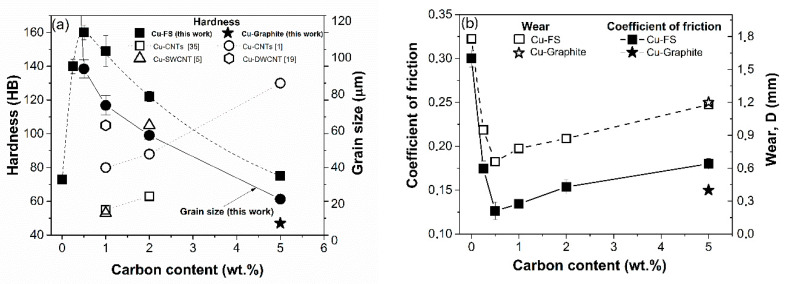
Hardness and grain size (**a**), friction coefficient and wear (**b**) in dependence on carbon concentration.

Hardness, grain size, and the tribological characteristics of the composites in dependence on FS content are shown in Figure 10. The dependence of the hardness has a maximum at 0.5 wt. % FS attaining a value of 160 ± 4 HB, which is higher than the majority of the data reported earlier for any Cu-C composites [1,2,3,4,5,19,20,21,22,23,24,25,26,27,28,29,30,31,32,33,34,35]. A further increase of FS concentration leads to the hardness falling down to 75 HB that may be explained by increase of Cu-boundaries quantity and appearance of soft FS agglomerates due to its inhomogeneous distribution. 

One of the strengthening mechanisms is hindering the dislocation movement by disperse carbon particles according to the Orowan mechanism. On the other hand, during a ball milled process, the material undergoes severe plastic deformation resulting in the distortion and fragmentation of the grain structure and the rise of dislocation density. Thus, the elastic stress, and dislocation knots and clusters became numerous and effective barriers for dislocation movement. Only work hardening was observed in the pure copper sample, prepared by the same technology as the composite ones; hardness of the sample reached 72 HB, which is 60% higher than that for pure annealed copper (about 40–45 HB), but significantly lower than that for Cu-(0.25 ÷ 2) wt. % FS composites. It can be assumed that disperse FS particles linked to the Cu matrix via an interphase Cu-O-C complex impedes Cu self-diffusion and prevents matrix weakening caused by grain growth, stress relaxation, and dislocation annihilation during hot pressing. Thus, dispersion strengthening, grain refinement, work, and strain hardening are the main strengthening mechanisms in the considered materials. The drawback of these materials is low plasticity, which is the consequence of high structural stress. 

Thermal conductivity of the composites is shown in Table 1.

Reference thermal conductivity of pure copper is 401 W/m*K at 300 K [36]. The thermal conductivity of our sample prepared out of pure copper amounts to only 332 W/m*K, because of micropores, structural defects, and oxides at the grain boundaries. The thermal conductivity continuously decreased with carbon content. The introduction of 0.25 and 0.5 wt. % FS leads to a decrease down to 320 and 288 W/m*K, respectively. Furthermore, the thermal conductivity continues to fall, in spite of an increase of recrystallized copper particles, as shown in Figure 6. This can be explained by defects, large amounts of interfaces, and lots of amorphous carbon, and, as a consequence, deficiency of copper-copper contacts. 

It is known that graphite and graphite-like carbon reduce the friction coefficient due to the formation of a thin lubrication layer between the counter-body and the material surface, reducing metal to metal contact. Interestingly, that in contrast to Cu-graphite composites, concentration dependency of the friction coefficient for Cu-FS composites has a minimum at 0.5 wt. %, coinciding with the hardness maximum and relatively high thermal conductivity. Further increase of FS content over 0.5 wt. % leads to the ascension friction coefficient. A similar tendency was observed in [24,25]; the ascending tendency may be a result of increasing porosity and structural inhomogeneity.

## 4. Conclusions

Cu–FS composites were fabricated using powder technology out of Cu powder and 0.25–5.00 wt. % fullerene soot. The size of the composite particles after milling decreases with an increase of FS concentration. In the process of ball milling, carbon does not diffuse to copper lattice, but distributes between its layers. Functional groups of FS promote the better adsorption of FS particles on the surface of copper and, as a result, lead to their better distribution in the composite. During hot pressing, FS reduces surface copper oxide with a formation of a gaseous product. Therefore, small additions of FS (0.25–0.5%) improve sintering of the composites, but higher concentrations worsen it. Copper reduction and recrystallization resulted in the “marble” pattern of the composite microstructure. The dependence of hardness on FS content has a pronounced maximum at 0.5 wt. % (160 HB). FS addition was advantageous for wear resistance and friction coefficient reduction.

## Figures and Tables

**Table 1:** Thermal conductivity of the composite materials.

	Cu-0%C	Cu-0.25%C	Cu-0.5%C	Cu-1%C	Cu-2%C	Cu-5%C
Thermal conductivity, W/m*K	332	320	288	227	193	113
